# Systematics and Plastome Evolution in Schizaeaceae

**DOI:** 10.3389/fpls.2022.885501

**Published:** 2022-07-13

**Authors:** Bing-Feng Ke, Goang-Jiun Wang, Paulo H. Labiak, Germinal Rouhan, Cheng-Wei Chen, Lara D. Shepherd, Daniel J. Ohlsen, Matthew A. M. Renner, Kenneth G. Karol, Fay-Wei Li, Li-Yaung Kuo

**Affiliations:** ^1^Institute of Molecular and Cellular Biology, National Tsing Hua University, Hsinchu, Taiwan; ^2^Taiwan Semiconductor Manufacturing Company, Hsinchu, Taiwan; ^3^Depto. de Botânica, Universidade Federal do Paraná, Curitiba, Brazil; ^4^Institut de Systématique, Evolution, Biodiversité (ISYEB), Muséum National d’Histoire Naturelle, EPHE, UA, CNRS, Sorbonne Université, Paris, France; ^5^Department of Biology, University of Florida, Gainesville, FL, United States; ^6^Department of Life Science, Biodiversity Program, Taiwan International Graduate Program, Biodiversity Research Center, Academia Sinica and National Taiwan Normal University, Taipei, Taiwan; ^7^Museum of New Zealand Te Papa Tongarewa, Wellington, New Zealand; ^8^Royal Botanic Gardens, South Yarra, VIC, Australia; ^9^Wildland Consultants, Rotorua, New Zealand; ^10^The Lewis B. and Dorothy Cullman Program for Molecular Systematics, New York Botanical Garden, Bronx, NY, United States; ^11^Boyce Thompson Institute, Ithaca, NY, United States; ^12^Plant Biology Section, Cornell University, Ithaca, NY, United States

**Keywords:** *chl*, *Microschizaea*, mycoheterotrophy, *ndh*, phyloplastome, Schizaeaceae

## Abstract

While the family Schizaeaceae (Schizaeales) represents only about 0.4% of the extant fern species diversity, it differs from other ferns greatly in gross morphologies, niche preferences, and life histories. One of the most notable features in this family is its mycoheterotrophic life style in the gametophytic stage, which appears to be associated with extensive losses of plastid genes. However, the limited number of sequenced plastomes, and the lack of a well-resolved phylogenetic framework of Schizaeaceae, makes it difficult to gain any further insight. Here, with a comprehensive sampling of ~77% of the species diversity of this family, we first inferred a plastid phylogeny of Schizaeaceae using three DNA regions. To resolve the deep relationships within this family, we then reconstructed a plastome-based phylogeny focusing on a selection of representatives that covered all the major clades. From this phylogenomic backbone, we traced the evolutionary histories of plastid genes and examined whether gene losses were associated with the evolution of gametophytic mycoheterotrophy. Our results reveal that extant Schizaeaceae is comprised of four major clades—*Microschizaea*, *Actinostachys*, *Schizaea*, and *Schizaea pusilla*. The loss of all plastid NADH-like dehydrogenase (*ndh*) genes was confirmed to have occurred in the ancestor of extant Schizaeaceae, which coincides with the evolution of mycoheterotrophy in this family. For chlorophyll biosynthesis genes (*chl*), the losses were interpreted as convergent in Schizaeaceae, and found not only in *Actinostachys*, a clade producing achlorophyllous gametophytes, but also in *S. pusilla* with chlorophyllous gametophytes. In addition, we discovered a previously undescribed but phylogenetically distinct species hidden in the *Schizaea dichotoma* complex and provided a taxonomic treatment and morphological diagnostics for this new species—*Schizaea medusa*. Finally, our phylogenetic results suggest that the current PPG I circumscription of *Schizaea* is non-monophyletic, and we therefore proposed a three-genus classification moving a subset of *Schizaea* species *sensu* PPG I to a third genus—*Microschizaea*.

## Introduction

Among the ~ 11,000 extant fern species, fewer than 40 belong to the family Schizaeaceae (Schizaeales; PPG I, 2016). Schizaeaceae differ from other ferns in their gross morphologies, niche preferences, life forms, and plastome structures (Reed, 1947; Bierhorst, 1971b; Kramer, 1990; Labiak and Karol, 2017). Despite the simplified morphology of these ferns, some foliar features can be used to separate them into different groups or genera—*Microschizaea*, *Actinostachys*, and *Schizaea* (Reed, 1947). These genera also differ in the unusual growth forms and habits of their gametophytes (Table 1). In *Microschizaea*, gametophytes are chlorophyllous, filamentous, and live on the soil surface (Table 1), and likely rely on fungal symbionts to survive in nutrition-poor habitats, such as bogs (Swatzell et al., 1996). By contrast, the genera *Actinostachys* and *Schizaea* have achlorophyllous, subterranean, endomycorrhizal gametophytes, with *Actinostachys* having tuberous and *Schizaea* thin-cylindrical growth forms (Table 1). The non-green and endomycorrhizal habit implies obligate mycoheterotrophy throughout their gametophyte generation, and might be an adaptation to deeply shaded habitats such as forest interiors, as well as sandy, nutrient poor soils (Graham et al., 2017). In comparison, the other members in the order Schizaeales—Lygodiaceae and Anemiaceae—have green planar and winged gametophytes typical of most ferns.

**Table 1:** Morphological comparison between the clades and genera of Schizaeaceae.

Clades in this study	*Microschizaea*	*Actinostachys*	*S. pusilla*	*Schizaea*
Genera *sensu* Reed (1947) (the species if containing the generic type)	*Microschizaea* (*M. fistulosa*)	*Actinostachys* (*A. digitata*)	*Microschizaea*	*Schizaea* (*S. dichotoma*)
Distribution	South America, New Zealand, Australia, South Pacific, Hawaii, Southeast Asia, Africa, Madagascar	South America, Australia, South Pacific, Asia, Madagascar	America	South America, New Zealand, Australia, South Pacific, Asia, Africa, Madagascar
Blade (Tryon and Lugardon, 1990)	Simple	Simple	Simple	Simple, dichotomous, or flabellate
Sporangiophores	Pinnate	Digitate	Pinnate	Pinnate
Sporangia	Two-rowed	Two- or four-rowed	Two-rowed	Two-rowed
Hairs among sporangia^1^	Absent	Absent or present	Present	Present
Spores	Subglobose to ovoid	Bilateral	Bilateral	Bilateral
P:E of spores	0.74–0.76	0.57–0.71	0.66	0.57–0.66
Perines^2^	Smooth, or grained	Smooth, seriate, pitted, or grained	Pitted	Smooth, pitted, or grained
Gametophytes	Green, surface-living, filamentous^3^	Non-green, subterranean, tuberous^4^	Green, surface-living, but with non-green and subterranean parts, filamentous^5^	Non-green, subterranean, thin cylinderic^6^? green and surface-living^7^

Information is mainly based on Reed (1947) and supplemented with other studies as noted.

1 Morphological observation from this study.

2 Tryon and Lugardon (1990), Parkinson (1994), Giacosa et al. (2015), and Giacosa and Barakat (2018).

3 Based on descriptions of species M. fistulosa, M. robusta, and M. rupestris (Goebel, 1915; Lash, 1966; Bierhorst, 1968).

4 Based on descriptions of species A. pennula, A. wagneri, A. digitata, A. spirophylla, A. germani, A. pennicilata, A. oligostachy, A. melanesica, A. macrofunda, A. intermedia, A. laevigata, and A. minuta (Bierhorst, 1965, 1966, 1968, 1971a, 1975; Amoroso et al., 2020).

5 Based on descriptions of species *S. pusilla* (Britton and Taylor, 1901; Swatzell et al., 1996).

6 Based on descriptions of species *S. dichotoma*, *S. elegans*, and *S. pseudodichotoma* (Bierhorst, 1967, 1968, 1971a, 1975).

7 Based on descriptions of species *S. bifida* (Thomas, 1902).

The current generic classification within Schizaeaceae has been largely based on morphological features (Reed, 1947; Tryon and Tryon, 1982), but whether these groupings reflect the phylogenetic relationships is unclear. The only molecular phylogenetics to date on Schizaeaceae was done by Wikström et al. (2002). In this study, while the plastid phylogeny was largely congruent with the morphology, their sampling included only one *Microschizaea,* the New World species *M. pusilla* (= *Schizaea pusilla*), which grouped with *Schizaea. Microschizaea* from the Old World (Table 1), including the type of the genus (*M. fistulosa*), have not yet been included in any molecular phylogenetic studies. The current phylogenetic consensus, in which *Microschizaea* is thought to be sister to *Schizaea*, was the basis for the acceptance of two genera in Schizaeaceae by Smith et al. (2006) and PPG I (2016) that include *Actinostachys* and *Schizaea*, under which *Microschizaea* is synonymized.

Recent studies on Schizaeaceae plastomes identified extensive gene losses in *Schizaea* and *Actinostachys*, which is unprecedented in ferns (Labiak and Karol, 2017; reviewed in Kuo et al., 2018a). For instance, all NADH-like dehydrogenase (*ndh*) genes were missing in all plastomes. These genes encode subunits for the NADH-like dehydrogenase complex that mediates photosynthetic electron flow of the photosystem I (Yamori and Shikanai, 2016), and is believed to be able to alleviate photo-oxidative stresses when plants are exposed to excessive light (Graham et al., 2017). In addition, all chlorophyll biosynthesis (*chl*) genes were missing in the *Actinostachys* plastome. The *chl* genes encode light-independent protochlorophyllide oxidoreductase (DPOR), and regulate one of the most important pathways of chlorophyll synthesis (Suzuki et al., 1998). Although similar losses have been documented in flowering plants that are predominately heterotrophs (Graham et al., 2017), no fern other than Schizaeaceae is known to have such extensive gene losses. It is possible that the plastid gene losses are associated with the mycoheterotrophic gametophytes in some Schizaeaceae members. To further test this relationship, it is necessary to look into the plastome of *Microschizaea*, which produces chlorophyllous gametophytes instead. In addition, the losses of some tRNA genes and structural changes in Schizaeaceae plastomes warrant further investigations with a more comprehensive species sampling.

In this study, we first inferred a Schizaeaceae phylogeny using a three-plastid-region dataset. Our sampling is the most comprehensive to date at the species level, and included all previously recognized genera and species groups (Table 1). Based on this phylogeny, representatives from every clade were then selected for a phyloplastomic reconstruction together with species from the other two Schizaeales families. This plastome-based approach resulted in better resolved inter-generic relationships within Schizaeaceae, allowing us to trace the evolutionary changes of plastome structures. To check whether *ndh* and *chl* genes have been transferred to the nuclear genome, we also examined their presence in transcriptomes. Finally, by mapping the gene loss events onto the phylogeny, we discussed the potential links to the specialization of life form in Schizaeaceae, in particular gametophytic mycoheterotrophy.

## Materials and Methods

### Sampling and Sequencing for Phylogenetic Analyses

A total of 47 Schizaeaceae specimens from 27 species (~ 77% of the species diversity of the family; PPG I, 2016) were sampled, including seven *Microschizaea* spp., seven *Actinostachys* spp., and 13 *Schizaea* spp. (Supplementary Table 1). This sampling covered all bioregions in each genus/clade (Table 1). For outgroups, we sampled the other two Schizaeales families: *Anemia phylliditis* from Anemiaceae and *Lygodium japonicum* from Lygodiaceae (PPG I, 2016). A modified CTAB protocol was used for DNA extractions (Kuo, 2015). The *rpoC2*, *rbcL*, and *trnL-L-F* (*trnL* gene + *trnL-F* intergenic spacer) were sequenced for our three-plastid-region dataset. PCRs were performed in 15 μl reactions each with 20 ng of genomic DNA, 0.5 μM of each primer, and 1 × SuperRed PCR Master Mix RED (TOOLS, New Taipei City, Taiwan). The resulting PCR products were purified and sequenced using the standard Sanger method with Applied Biosystems 3730XL (Thermo Fisher Scientific, Waltham, MA, United States of America) at Genomics Corp. (New Taipei City, Taiwan).

For the plastome phylogeny, we selected both Schizaeales outgroups and a total of 11 Schizaeaceae representatives. These included species from every genus and major clade within Schizaeaceae (two *Microschizaea* spp., two *Actinostachys* spp., and seven *Schizaea* spp.; Supplementary Table 1). Among these, published plastome sequences were already available for *L. japonicum* and three of the 11 species of Schizaeaceae (Gao et al., 2013; Labiak and Karol, 2017), and these were used for all downstream analyses. To assemble the remaining plastomes, we used the Illumina reads generated by either the GoFlag project (Breinholt et al., 2021) or the genome skimming of this study. When the sequencing depth of a GoFlag sample was insufficient to yield a circular plastome using NOVOplasty (see below for details), we designed PCR primers and closed the gaps between the contigs. The PCR recipe was the same as described earlier. For the genome skimming, we first sheared the genomic DNA into 400 ~ 500 bp fragments using a Covaris S2 ultrasonicator (Covaris, Woburn, MA, United States of America), which were then input into the NEBNext Ultra II DNA Library Prep Kit (New England Biolabs, Ipswich, MA, United States of America). Sequencing was done by HiSeq X Ten (Illumina, San Diego, CA, United States of America) with 150 bp PE and ~ 3Gb per sample. Fastp (Chen et al., 2018) was used to trim the reads using the default settings. NOVOplasty (Dierckxsens et al., 2017) was used to assemble the plastomes with the setting of “Kmer = 39,” and conspecific *rbcL* sequences were used as the input seeds. These plastome assemblies were annotated using Geneious (Kearse et al., 2012) with the published Schizaeaceae plastomes (GenBank accessions: KU764518, KX258660*-*61) as references. We manually inspected every protein-coding gene annotation, and adjusted the coordinates if necessary.

Details about the PCR primers are provided in Supplementary Table 2. Details about the voucher information, GenBank accessions, and NCBI Sequence Read Archive accessions can be found in Supplementary Table 1.

### Phylogenetic Analyses

For the three-plastid-region dataset (i.e., “*rpoC2* + *rbcL* + *trnL-L-F*”), the nucleotide sequences were first aligned with MUSCLE (Edgar, 2004), as implemented in AliView (Larsson, 2014), and then concatenated into a single matrix. This three-plastid-region matrix was partitioned by gene, by intergenic spacer (IGS), and by codon position in order to find the best partition scheme and substitution models using ModelFinder (Kalyaanamoorthy et al., 2017) with the Bayesian information criterion (BIC; Schwarz, 1978). Based on the inferred partition scheme and the substitution models, IQtree 1.6.8 (Nguyen et al., 2015) was used to construct the maximum likelihood (ML) phylogeny with 1,000 ultrafast bootstrap replicates (UFBS). The Bayesian phylogeny was inferred using MrBayes 3.2.7 (Ronquist et al., 2012). Two simultaneous runs were carried out with four chains (10 million generations each). Each chain was sampled every 1,000 generations. Log likelihoods of MCMC runs were inspected in Tracer 1.6 (Rambaut and Drummond, 2013) and RWTY (Warren et al., 2017) to confirm their convergence. The first 25% of the sample was discarded as burn-in, and the remaining was used to calculate the maximum clade credibility consensus tree with TreeAnnotator (Rambaut and Drummond, 2013).

For the phyloplastomic datasets, we only included coding sequences, introns, and intergenic spacers (IGS) that were consistently located in the large single copy (LSC) region of the Schizaeaceae plastomes. The restriction to LSC genes aimed to reduce phylogenetic artifacts resulting from substitution rate heterotachy, because the movement of genes between the inverted repeat (IR) and single copy (SC) regions would lead to changes in their substitution rates (Li et al., 2016). For alignment of every individual DNA region, we used MAFFT v7.450 (Katoh and Standley, 2013) and MACSE v2.03 (Ranwez et al., 2011). In total, we compiled five different matrices that consist of either the coding genes, noncoding regions (i.e., “IGS/INTRON”), or both (i.e., “67CDS + IGS/INTRON”). In the three coding gene matrices, 67 loci from the LSC were included with sequences of either (1) the first two codon positions (i.e., “codon1 + 2”), (2) the third codon position (i.e., “codon3”), and (3) all three codon positions (i.e., “67CDS”). For each of the five matrices, we performed four analyses with different models and partitions, as detailed in Table 2. With all the matrix/partition/model combinations, we conducted a total of 20 (= 5 × 4; Table 2) ML phylogenetic analyses in IQtree 1.6.8 each with 1,000 UFBS replicates and a series of tree topology tests (such as KH, SH, ELW, and AU; detailed in Minh et al., 2020) with a RELL replicate number of 10,000.

**Table 2:** Summary of phylogenetic analyses in this study.

Matrix	Prior partition	Final partition	Model	UFBS branch support^1^				Major topology^2^	Excluded topology^2^^,^^3^
				Node A	Node B	Node C	Node D		
67CDS + IGS/INTRON	By gene by codon positions; by spacer/intron	Inferred by ModelFinder	Inferred by ModelFinder	96	89	–	–	I	II
67CDS + IGS/INTRON	By gene by codon positions; by spacer/intron	By gene by codon positions; spacer/intron	GTR + F + R10	92	91	–	–	I	None
67CDS + IGS/INTRON	n.a.	n.a.	GTR + FO*H4	97	92	–	–	I	II
67CDS + IGS/INTRON	No	No	GTR + F + R10	77	99	–	-	I	II
67CDS	By gene by codon positions	Inferred by ModelFinder	Inferred by ModelFinder	98	52	–	–	I	None
67CDS	By gene by codon positions	By gene by codon positions	GTR + F + R10	96	–	50	–	II	None
67CDS	n.a.	n.a.	GTR + FO*H4	99	–	68	–	II	II
67CDS	No	No	GTR + F + R10	98	60	–	–	I	III
codon1 + 2	By gene	Inferred by ModelFinder	Inferred by ModelFinder	56	69	–	–	I	I, II
codon1 + 2	By gene	By gene	GTR + F + R10	57	69	–	–	I	None
codon1 + 2	n.a.	n.a.	GTR + FO*H4	37	46	–	–	I	I, II
codon1 + 2	No	No	GTR + F + R10	-	58	–	46	III	None
codon3	By gene	Inferred by ModelFinder	Inferred by ModelFinder	99	–	77	–	II	III
codon3	By gene	By gene	GTR + F + R10	99	–	92	–	II	III
codon3	n.a.	n.a.	GTR + FO*H4	98	–	61	–	II	None
codon3	No	No	GTR + F + R10	98	–	74	–	II	III
IGS/INTRON	By spacer/intron	Inferred by ModelFinder	Inferred by ModelFinder	–	96	–	61	III	II
IGS/INTRON	By spacer/intron	By spacer/intron	GTR + F + R10	–	96	–	77	III	II
IGS/INTRON	n.a.	n.a.	GTR + FO*H4	–	98	–	65	III	II
IGS/INTRON	No	No	GTR + F + R10	–	98	–	58	III	II
*rpoC2 + rbcL + trnL-L-F*	By gene by codon positions; spacer/intron	Inferred by ModelFinder	Inferred by ModelFinder	–	61	–	50	III	n.a.

1 Showing only values from the major topology.

2 The topologies in Figure 2.

3 The significantly (*p* < 0.05) excluded topology inferred by one of these methods: bp-RELL, p-KH, p-SH, p-WKH, p-WSH, c-ELW, and p-AU, detailed in “Topology Tests” of Minh et al. (2020).

To examine the potential effects of homoplasy driven by rapidly evolving sites in CDS, particularly the third codon positions, we also analyzed two degenerate-coded matrices: one “67CDS” with “Degen”-coding (Zwick et al., 2012) and one “codon3” with RY-coding of third codon positions, and compared these results with those generated from original non-degenerated ones. For these two matrices, the same region partitions and model selection were used as those inferred by the original non-degenerated matrices, and the ML phylogenetic analyses was then performed with 1,000 UFBS replicates in IQtree 1.6.8. In addition, to examine the evolution of Schizaeales *chl* genes in more detail, we combined their sequences with those used in Kuo et al. (2018a), which has a complete order-level sampling. These sequences were aligned with MAFFT, and used to infer an ML phylogeny in IQtree 1.6.8 with the model GTR + FO*H4 and 1,000 UFBS replicates.

### Morphological Comparisons Within the *Schizaea dichotoma* Complex

We examined herbarium collections from MO, P, TAIF, UC and WELT to conduct morphological comparisons between the *S. dichotoma* complex from Africa/Malagasy and Asia/Oceania. The Asian/Oceanian *S. dichotoma* complex contains several species—*S. asperula*, *S. bifida*, *S. biroi*, *S. dichotoma* and *S. forsteri—*whereas the African/Malagasy complex was represented by a single species—*S. medusa* sp. nov. We also measured several quantitative traits from these specimens, such as rhizome thickness, frond sizes, and stipe sizes (Supplementary Table 3).

### Confirming the Existence of *ndh* and Missing Plastid Genes in Transcriptomes and Genomes

To confirm whether the nuclear-encoded *ndh* complex related genes was present or not in the Schizaeales, we downloaded their homologue sequences from the well-annotated case of *Apostasia odorata* (detailed in the Supplementary Material S2 of Lin et al., 2017), and blast-searched them against six Schizaeales transcriptomes (Qi et al., 2018; Shen et al., 2018; One Thousand Plant Transcriptomes (OTPT) Initiative, 2019) and two fern whole genomes (Li et al., 2018). For *PnsB1*, the homologue sequence from *Marchantia polymorpha* (Ueda et al., 2012) was used instead, because the annotated sequence from *A. odorata* (GenBank accession: KX156894) appeared to be misidentified. We also included the *ndhV* from *Zostera marina* (Ma et al., 2022), because the sequence of *A. odorata* appeared to be unusually diverged from other plants. The transcriptomes were obtained from previous studies which used foliar, photosynthetic tissues for RNAseq, and included two Lygodiaceae (*Lygodium japonicum* and *Lygodium flexuosum*), two Anemiaceae (*Anemia tomentosa* and *Anemia phyllitidis*), and two Schizaeaceae (*Actinostachys digitata* and *Schizaea dichotoma*). We first used the sequences from *A. odorata*, *Z. marina*, and *M. polymorpha* as the query for tBLASTx searches against the *Azolla filiculoides* and *Salvinia cucullata* genomes *via* FernBase (https://www.fernbase.org/). Based on the criterion of a percentage of identity, we obtained the best-matched sequences from the fern genomes. To confirm whether these sequences are homologues or not, we conducted tBLASTx searches against the nucleotide collections of NCBI, and inferred their gene trees based on the blast results. After obtaining the fern homologous sequences from these genomes, we then added these sequences into the tBLASTx searches against the transcriptomic assemblies using blast-2.10.0 + (Camacho et al., 2009). Only the transcripts having > 60% sequence identity and consistent hits to all query homologues (i.e., those from the two ferns, *A. odorata*, *Z. marina*, and *M. polymorpha*) were kept.

To examine the possibility that the missing protein-coding genes in the plastome have been transferred to and are now transcribed in the nuclear genome, we also conducted local tBLASTx searches for these genes as mentioned above. The query sequences were derived from plastomes of the closest relatives that still retain those genes (e.g., using Anemiaceae’s *rps16* sequence to blast against the Schizaeaceae’s transcriptomes).

## Results

### Plastome Features of Schizaeaceae

**Figure 1:**
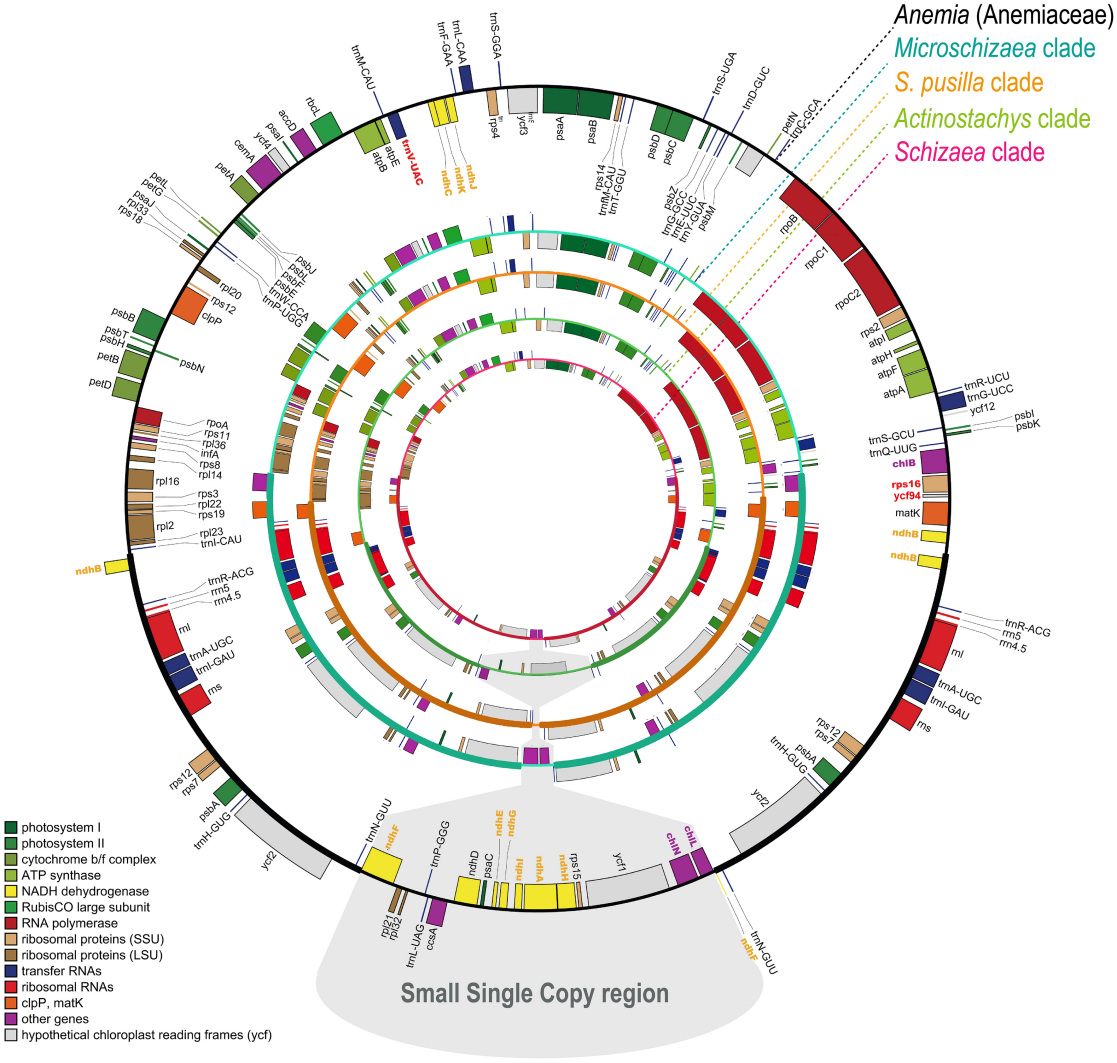
The plastome maps of Schizaeaceae and Anemiaceae. The genes missing in Schizaeaceae plastomes have bolded and colored names. The maps are derived from OrganellarGenomeDRAW (Greiner et al., 2019).

**Figure 2:**
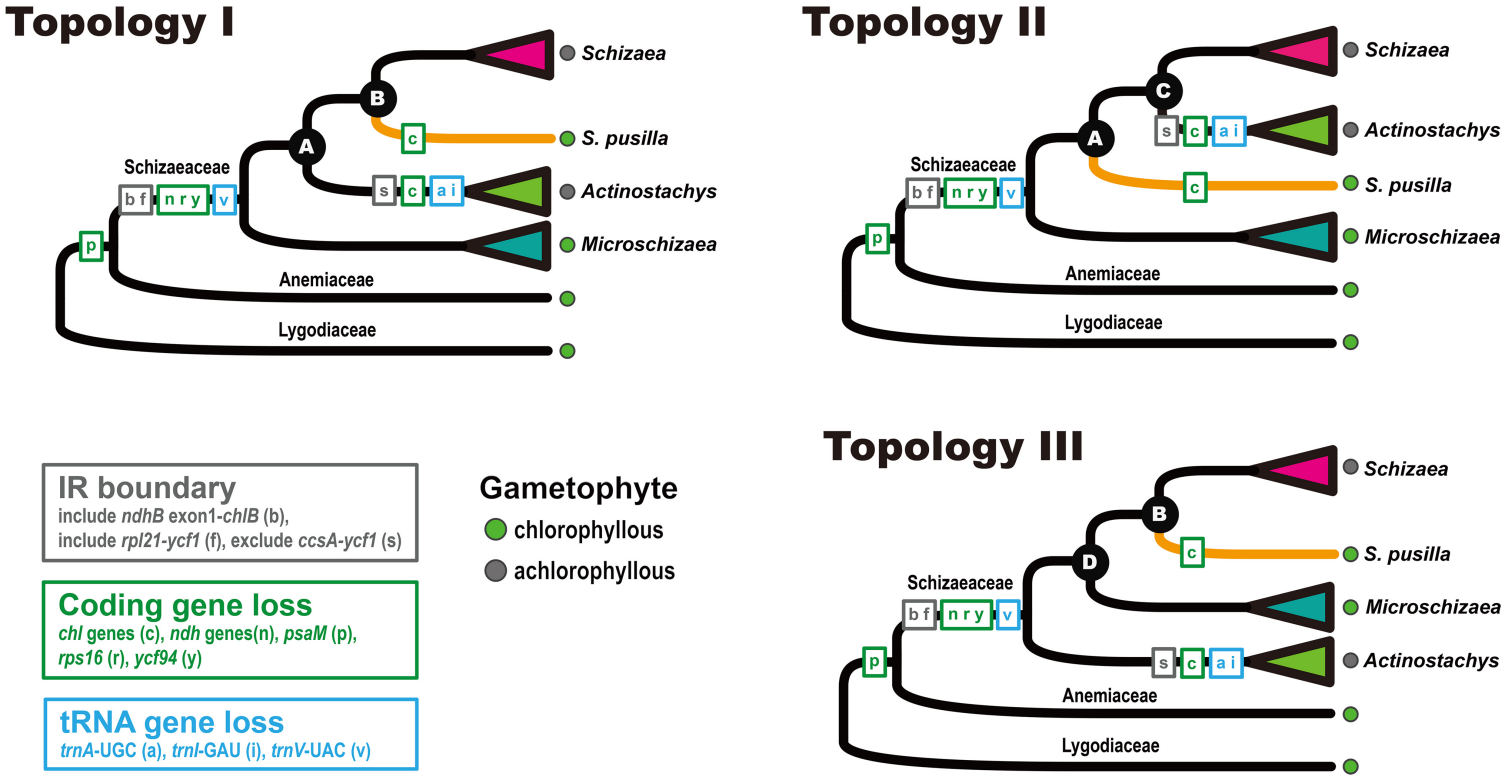
Plastome structural evolution in Schizaeaceae. The three topologies were derived from the phyloplastomic analyses that included different data matrices, models, and partitions.

In total, nine plastomes were assembled in this study, including one *Anemia* and eight Schizaeaceae. They all assembled into a single circular contig, except for the plastome of *Microschizaea tenella* in which the gap between *psbK* and *trnQ* could not be closed by PCR and was thus separated into two contigs. All members of Schizaeaceae share several plastome features, including the expansions of the IR and losses of all *ndh* genes (*ndhA–J*), *rps16*, *ycf94*, and several tRNA genes (Figure 1). IR/SC boundaries of the Schizaeaceae plastomes are almost identical, and their small single copy region (SSC) are highly reduced (Figure 1). In the most extreme case, the SSC of *S. pusilla* is only 1,091 bp in length and contains only one gene, *trnN*. *Actinostachys*, on the other hand, appears to be an outlier where the SSC expanded to include *ccsA*, *rps15*, and *ycf1*. The *chl* genes (*chlB*, *chlN*, and *chlL*) are absent in the plastomes of two clades—*S. pusilla* and *Actinostachys*, but this is unlikely to have resulted from a single loss event (Figure 2; see below in “Discussion”). The loss of *psaM* gene was shared by Schizaeaceae and its sister family—Anemiaceae (Figure 2).

### Phylogeny of Schizaeaceae

**Figure 3:**
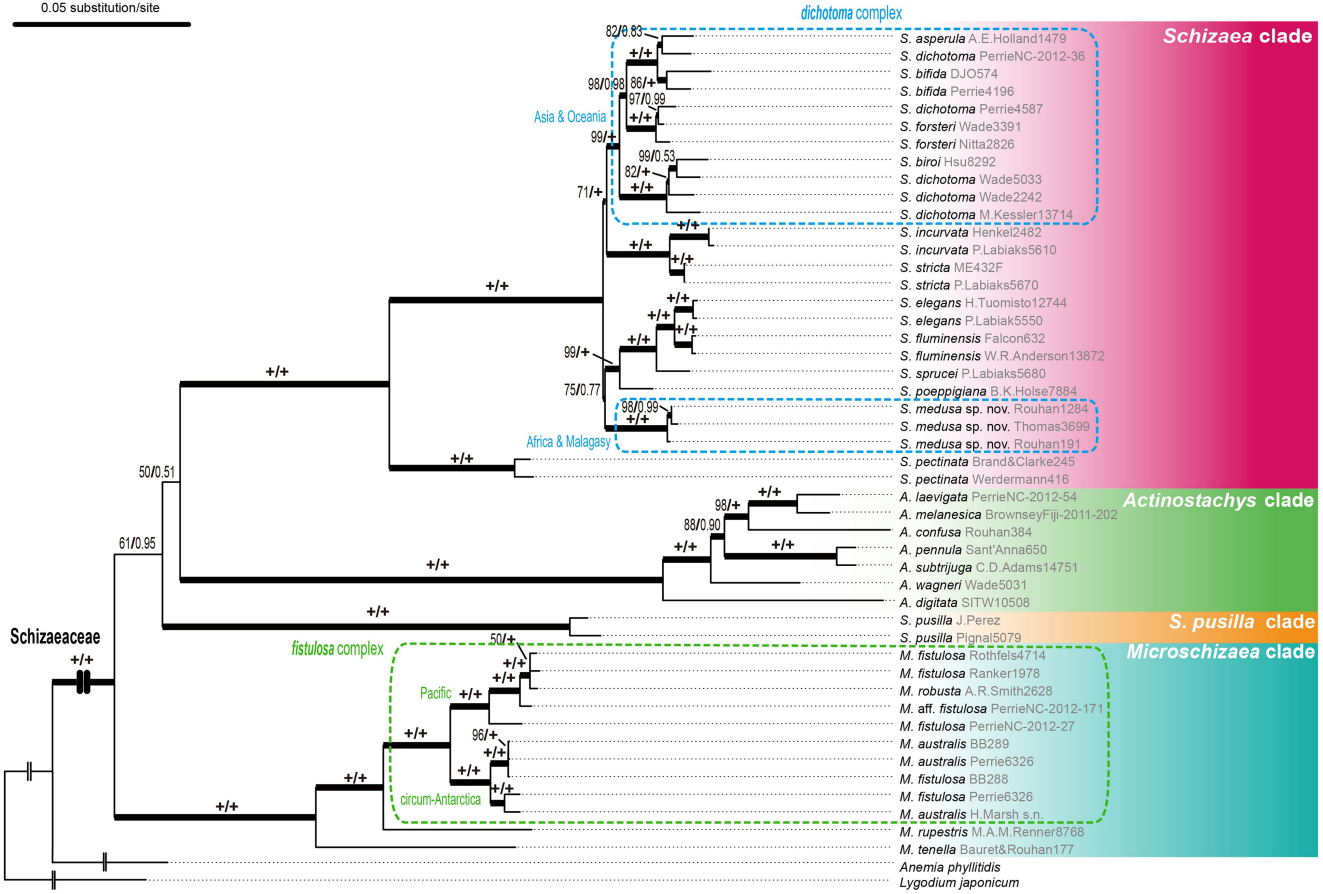
The plastid phylogeny of Schizaeaceae based on the *rpoC2* + *rbcL* + *trnL-L-F* dataset. Maximum likelihood ultrafast bootstrap supports (UFBS) and Bayesian inference (BI) are indicated on each branch as UFBS/BI.

From our three-plastid-region and plastome datasets (Figures 2, 3) four well-supported clades were recovered (UFBS = 100, Bayesian inference = 1.00) within Schizaeaceae—*Schizaea*, *S. pusilla*, *Actinostachys*, and *Microschizaea* (Figure 3). These clades closely matched their generic definition by Reed (1947), except for *S. pusilla* which formed a rather distinct clade from other *Microschizaea* spp. However, the inferred inter-clade relationships differed among our phylogenetic analyses (Table 2). Generally, topology I received the highest branch supports, and another two were rejected by most of our topological tests (Table 2). Only the “codon3” matrix with a gene-partitioned GTR + F + R10 model highly supported topology II (Table 2), but this support was declined or nearly unchanged when applying the degenerate-coded matrices with analyses (Supplementary Table 4).

In the *Schizaea* clade, the *S. dichotoma* complex was non-monophyletic with two separate lineages, one from Africa/Madagascar, and the second from Asia/Oceania (Figure 3). In the *Microschizaea* clade, *M. robusta* and *M. australis* were nested within *M. fistulosa,* rendering *M. fistulosa* polyphyletic (Figure 3).

### Presence of *ndh* and *chl* Genes in Transcriptomes and Genomes

**Figure 4:**
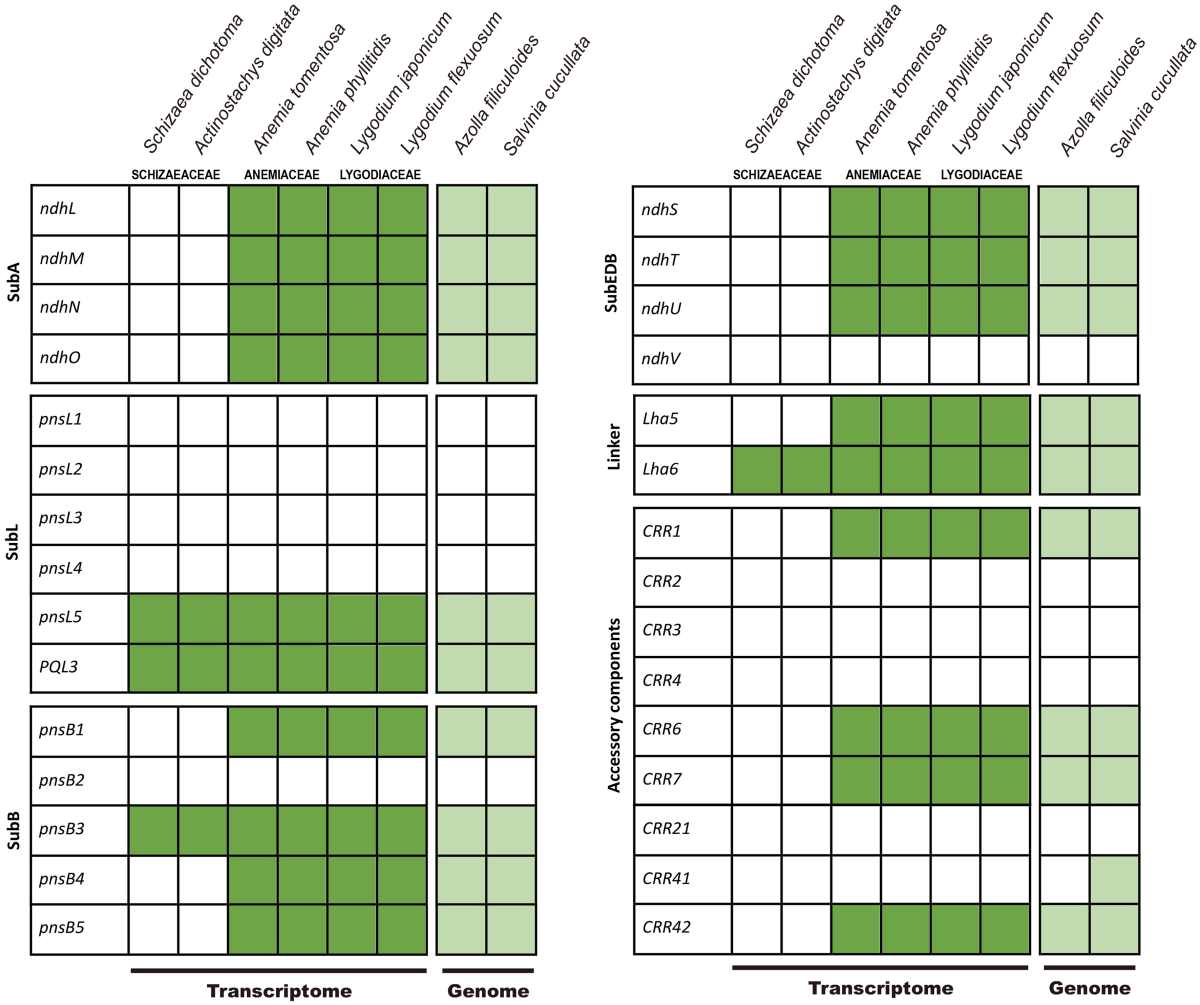
Presence of the nuclear-encoded genes of NADH-like dehydrogenase (*ndh*) complex in Schizaeales transcriptome and fern genomes. The subcomplexes (sub), linker, and accessory components follow the scheme by Shikanai (2016).

We detected the nuclear-encoded *ndh*-related genes in the transcriptomes of other Schizaeales (Anemiaceae and Lygodiaceae) and the genomes of *Salvinia* and *Azolla*, except for *pnsL1*–*4*, *psnB2, ndhV, CRR2*–*4*, and *CRR21* (Figure 4). For *CRR41*, we identified it in *Salvinia* but not in *Azolla*. This gene likely existed in other Schizaeales (Anemiaceae and Lygodiaceae), whose *CRR41*-matched transcripts showed consistent blast-hits to the homologous queries of *CRR41* although relatively low in the identity ranging from 40–54%. In contrast, all plastid and most nuclear-encoded *ndh*-related genes were missing in the Schizaeaceae transcriptomes (Figure 4). We were also unable to detect any *chl* genes in the transcriptome of *Actinostachys*, whose plastomes also lack these genes. The plastid *rps16* and *ycf94* genes were also absent from the transcriptomes of Schizaeaceae, and *psaM* was absent from both Schizaeaceae and Anemiaceae.

## Taxonomic Treatment

**Figure 5:**
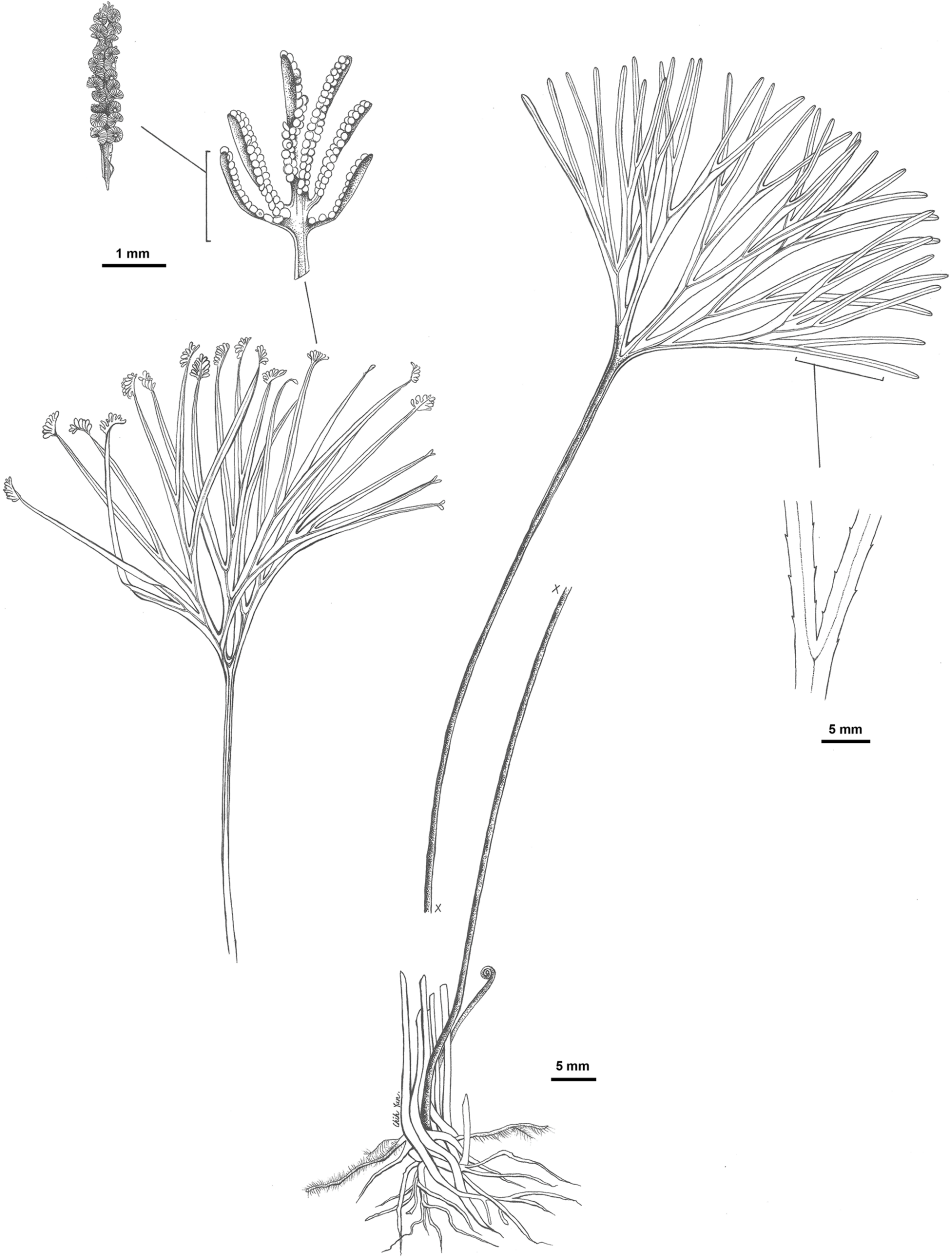
Illustration of *Schizaea medusa* L.-Y. Kuo, B.-F. Ke, F.-W. Li, and Rouhan, sp. nov., based on the holotype *G. Rouhan 1284* (P02432844).

***Schizaea medusa*** L.Y.Kuo, B.F.Ke, F.W.Li, and Rouhan, sp. nov (Figure 5).

Type: MADAGASCAR, Andapa, Sava, 31 October 2011, *G. Rouhan, M. Gaudeul* & *J. Ranaivo 1284* (holotype: P[P02432844]! isotypes: NY, TAN).

Diagnosis: This species is morphologically most similar to *S. dichotoma* and other members of this species complex, but, in comparison, has a thicker (usually 5–12 mm in diameter) and erect/ascending rhizome (vs. creeping rhizomes with 0.7–6 mm diameter in other members of the *S. dichotoma* complex). The blade-to-stipe ratio in this species is on average higher than that in other members of the *S. dichotoma* complex (Supplementary Figure 1).

Description: **Plants** terrestrial. **Rhizomes** erect or ascending, (3–) 5–12 mm in diameter, bearing chestnut-brown, septate hairs. **Fronds** 20.5–42.2 cm long; stipes 11.1–30.0 cm long; sterile portion of laminae 2–6 times dichotomously divided, 4.2–12.8 cm long, ±terete, furrowed on one side with stomata 81–114 μm long. Fertile portion of laminae pinnately divided, 2–11 mm long, 1–4 mm wide. **Fertile segments** in 4–15 pairs per spike, infolded, 1–5 mm long, with fimbriate margins. **Sporangia** borne in two rows, 24–26 per branch, intermixed with yellow-brown septate hairs. **Spores** with perines granular or smooth, polar lengths 22–30 μm, equatorial lengths 37–38 μm, ratios of polar to equatorial length 0.57–0.66.

Additional Specimens Examined: **Madagascar**. Tulear Province, 22 March 1992, *R. A. Clement, P.B. Phillipson* & *G. Rafamantanantsoa 2088* (MO); 16 March 1985, *L. J. Dorr 3979* (MO); Toamasina, 04 October 2003, *R. Razakamalala et al.* *773* (MO); 12 June 2004, *R. Ranaivojaona et al.* *750* (MO); 10 December 2006, *J. Razanatsoa* & *T. Marcellin 268* (MO); 1 February 2001, *F. Ratovoson et al.* *450* (MO); 24 October 2001, *J. -N. Labat 3409* (MO); 8 June 1987, *P. Phillipson 1879* (MO); 31, May 2007, *A. Razanatsima et al.* *265* (MO); 21 October 2007, *P. Antilahimena* & *T. Marcelin 5859* (MO); Antsiranana Province, 22 October 1989, *J.S. Miller* & *A. Randrianasolo 4343* (MO); 2 December 2007, *C. Rakotovao et al.* *3878* (MO); 19 January 2004, *L. Nusbaumer, LN 1044* (MO); 11 September 2007, *L. Nusbaumer et al., LN 2423* (MO); 8–12 May 1993, *S. Malcomber* & *C. Hemingway 2483* (MO); 26 November 2000, *A. Rasolohery 100* (MO); 11 November 2006, *C. Rakotovao et al.* *3326* (MO); 30 October 2000, *P. Antilahimenaet et al.* *622* (MO); 24 September 2004, *T. Janssen 2386* (P); Fianarantsoa Province, 26 August 2003, *D. Rabehevitra, R. Razakamalala* & *I. Dely 562* (MO); 4 June 1992, *R. Rakoto 58* (MO); 14 January 2006, *A. Anderberg et al.* *71* (MO); 10 October 1992, *H. van der Werff et al.* *12651* (MO); 30 September 1987, *P. Phillipson 2197* (MO); 12 October 2015, *G. Rouhan 1656* (P); Toliara, 25 May 2005, *N. M. Andrianjafy et al.* *1105* (MO); November 2005, *Richard Razakamalala et al.* *2523* (MO); 27 February 2009, *R. Razakamalala et al.* *4332* (MO); 20 November 2009, *C. Rakotovao et al.* *4647* (MO); 19 February 2009, *C. Rakotovao et al.* *4344* (MO); 13 March 1989, *N. Dumetz 574* (MO); Tamatave Province, 2 November 1985, *L. J. Dorr et al.* *4320* (MO); 2–5 November 1984, *L. J. Dorr* & *L. C. Barnett 3223* (MO); 29 August 1987, *G. E. Schatz* & *W. D’Arcy 1489* (MO); 19 January 1986, *L.C. Barnett* & *C. Rakotozafy 4588* (MO); Anosy region, 22 May 2006, *F. Randriatafika et al.* *675* (MO); 26 November 2004, *G. Rouhan 481* (P); Majunga Province, 11 January 1985, *A. Rakotozafy* & *R. Rajemisa 289* (MO); Mahajanga, 25 April 2007, *D. Ravelonarivo 226* (MO); Antananarivo, 16 November 2003, *P. P. Lowry II et al.* *6274* (MO); August 1987, *David K. Edelman 142* (MO); Lokobe Strict Reserve, 4 October 1991, *C. Birkinshaw 35* (MO); **Tanzania**. Morogoro region, 22 September 1984, *Thomas 3699* (MO, P); 2 December 1987, *J. Lovett* & *D. W. Thomas 2621* (MO); Iringa, 2 August 1989, *C. J. Kayombo 764* (MO). **Réunion**. Hauts de Saint Louis, 2006, *J. Dupont 3997* (P); 6 July 1973, *T. Cadet 4316* (P). **Mauritius**. Mare Longue Plateau, 1 April 2003, *G. Rouhan 191* (P).

Distribution: Africa (Tanzania), Madagascar, Réunion, Comoros, and Mauritius.

Etymology: The fronds of this species produce numerous terminal branches that are somewhat interlaced each ending with a sporangiate spike. This foliar diagnostic is superficially similar to Medusa, a well-known winged human female in Greek mythology, whose head is crowned by entwining snakes.

## Discussion

### A New Generic Classification of Schizaeaceae

Our phylogenetic analyses based on different datasets, models, and partitions resulted in three general topologies (Figure 2). While data from plastome structure and gene content failed to provide additional evidence, we have more confidence in topology I (Table 2). This topology received, on average, higher branch supports (i.e., nodes A and B), and was not rejected in most of the topology tests (Table 2). On the other hand, although topology II also received high branch supports in some analyses, it was mostly derived from matrices comprised of the third codon positions. It is likely that the support for topology II is a result of high homoplasy at the third codon position (Supplementary Table 4), which is known to have higher substitution rates and therefore be more likely to reach saturation (e.g., Breinholt and Kawahara, 2013).

Notably, our study is the first to include *Microschizaea* species other than *S. pusilla*. All the inferred phylogenies suggested that *Microschizaea sensu* Reed (1947) is not monophyletic and fell into two distinct, non-sister clades—*Microschizaea* and *S. pusilla* (Figure 2). The *Microschizaea* clade comprises all *Microschizaea* species except *S. pusilla* (Figures 2, 3). The position of *S. pusilla* varied across the three inferred topologies, but was never found to be sister to *Microschizaea*. In the most highly supported topology (topology I), *S. pusilla* was placed sister to the *Schizaea* clade similar to what was reported by Wikström et al. (2002). The close relationship between the *S. pusilla* and the *Schizaea* clades is morphologically supported by the shared presence of multicellular hairs intermixing with sporangia, a trait that is absent in the *Microschizaea* clade (Table 1). *Schizaea pusilla* also differs from *Microschizaea* by its more ellipsoidal (i.e., bilateral) spores with a ratio of polar to equatorial length (P:E) about 0.66, compared to the subglobose or ovoid spores in the *Microschizaea* clade with P:E around 0.72–0.76 (Reed, 1947; Table 1). Finally, spore perines of *S. pusilla* are alveolate with shallow pits but those of the *Microschizaea* clade are granular or smooth (Table 1).

Despite bearing morphological and phylogenetic differences, *Microschizaea* was retained in synonymy of the genus *Schizaea* in the latest phylogenetic classification of PPG I (2016), and *Actinostachys* was there recognized as the second genus of Schizaeaceae. Here, we show *Schizaea* as circumscribed by PPG I is very likely paraphyletic. We therefore propose a three-genus framework for Schizaeaceae that recognizes *Microschizaea*, *Actinostachys*, and *Schizaea*. This classification is similar but different from Reed (1947) in that we placed *S. pusilla* in *Schizaea* rather than in *Microschizaea*. It can be argued that given its unresolved placement and distinct morphology, *S. pusilla* could be erected as a separate genus. We are however hesitant to do so based on the current data. Regarding the infra-generic system of Reed (1947), many of his subgenera and (sub)sections are non-monophyletic; further studies are needed to provide a clear picture for these infra-generic schemes.

### Systematics of Species Complexes

The simple foliar structure in Schizaeaceae presents taxonomic challenges, particularly in the *Microschizaea fistulosa* and *Schizaea dichotoma* species complexes. The *M. fistulosa* complex contains the polyphyletic “*M. fistulosa*” and most of its congeneric members (Figure 3), and has a broad distribution across Southeastern Asia, Pacific Islands, Oceania, and South America (Lash, 1966; Brownsey and Perrie, 2014; Giacosa et al., 2015). Morphologically variable but continuous forms make taxonomy of this complex still unsettled. There are more than ten names within this complex but these names are always treated under a single name—*M. fistulosa* or with another—*M. australis* (Lash, 1966; Brownsey and Perrie, 2013, 2014). In addition, two cytotypes/ploidy levels have been discovered. The specimens in New Zealand can be separated into *M. australis* with a lower chromosome number (*n* = 94) and smaller overall plant sizes, and *M. fistulosa* with a higher chromosome number (*n* = c.150, 190) and larger individual sizes (Brownsey and Perrie, 2013). From our plastid tree, Pacific samples of this complex appear to form a group, while circum-Antarctic samples form another (Figure 3). However, because plastid sequences can track only the maternal lineage in ferns (reviewed in Kuo et al., 2018b), the current phylogeny is still insufficient to shed light into any polyploidization and reticulation history in the *M. fistulosa* complex. Future systematics studies need to incorporate cytological information, details of microcharacters, and analyses of nuclear markers.

The *S. dichotoma* complex likewise exhibits wide morphological and cytological variations (Brownsey and Perrie, 2014). Some forms have been formally named to reflect their distinct morphology, such as *S. asperula* showing sterile-fertile dimorphic fronds and *S. bifida* showing only twice-to-thrice bifurcated lamina. However, most members of this complex are poorly characterized, and have been collectively lumped into “*S. dichotoma*”, which is found to be polyphyletic in our phylogeny (Figure 3). We discovered that the African and Malagasy specimens form a clade that is phylogenetically distinct from the rest of the *S. dichotoma* members (Figure 3). Because the types of all the previously named species in this complex are based on Asian and Oceanian materials, a new species is warranted, which we named *Schizaea medusa*. By quantifying several key morphological characters, we showed that *S. medusa* can be distinguished from *S. dichotoma* (Supplementary Figure S1; also see in “Taxonomic Treatment”). There are likely additional cryptic species in the *S. dichotoma* complex and more studies are clearly needed.

### Specialization of Gametophytic Lifestyle and Losses of Plastid Genes

Gametophytes of Schizaeaceae are peculiar among extant ferns because of their strongly mycotrophic lifestyle, whether they are achlorophyllous (like *Schizaea* and *Actinostachys*) or not (like *S. pusilla* and *Microschizaea*). Only one exceptional case, reporting a surface-living and chlorophyllous gametophyte in the *Schizaea* clade, is from the observation of *S. bifida* by Thomas (1902). However, this record needs to be confirmed because *S. bifida* usually co-occurs with *Microschizaea* (Brownsey and Perrie, 2014), and fern gametophytes in the field could be easily misidentified without genetic evidence (Nitta and Chambers, 2022; Wu et al., 2022). Despite being chlorophyllous, gametophytes of *S. pusilla* and *Microschizaea* still show several properties that are not found in other chlorophyllous fern gametophytes. Their gametophytes are filamentous and partially or sometimes completely underground, with their subterranean parts being achlorophyllous (Britton and Taylor, 1901; Lash, 1966; Bierhorst, 1968). In addition, previous studies failed to regenerate sporophytes from these gametophytes under axenic conditions (Lash, 1966; Swatzell et al., 1996), implying that microbial symbionts might be required for sexual reproduction (Britton and Taylor, 1901; Lash, 1966; Swatzell et al., 1996). Finally, at least in *S. pusilla*, the gametophytes unusually exhibit a negative phototropism during spore germination (Kiss, 1994). Taken together, Schizaeaceae are clearly heterotrophic or mixotrophic at the gametophyte stage, and have intimate associations with fungal symbionts.

Heterotrophic or mixotrophic plants often display certain diagnostic genomic signatures (e.g., Vogel et al., 2018; Su et al., 2019; Xu et al., 2021), such as plastomes with extensive gene losses (Graham et al., 2017; Hadariová et al., 2018; Wicke and Naumann, 2018). Here we confirmed that the loss of plastid *ndh* genes is a synapomorphy of extant Schizaeaceae (Figure 2), and we also could not detect expression of most nuclear *ndh*-related genes from Schizaeaceae transcriptomes. In other words, there might be a concerted loss of *ndh*-related genes in both genomic compartments (Figure 4), a feature that has been suggested to be the initial (but irreversible) step toward mycoheterotrophy in flowering plants (Graham et al., 2017; Lin et al., 2017). Interestingly, a separate fern lineage *Stromatopteris* (Gleicheniaceae), which also produces mycoheterotrophic gametophytes (Bierhorst, 1971b), has likewise experienced *ndh* gene losses in the plastome (Du et al., 2022).

Loss of the plastid *chl* genes is frequently found in plants with a heterotrophic and/or achlorophyllous nature for their gametophyte generation (e.g., all flowering plants), and is thus considered as one genomic indicator for heterotrophs (Ueda et al., 2014). These plastid *chl* genes encode all subunits for light-independent protochlorophyllide oxidoreductase (DPOR), which plays an important role for chlorophyll synthesis under dark, particularly during the gametophyte stage (Suzuki et al., 1998; Ueda et al., 2014). Complete lack of *chl* genes had been documented in two fern lineages, Psilotaceae and *Actinostachys* in Schizaeaceae (Grewe et al., 2013; Zhong et al., 2014; Labiak and Karol, 2017; Kuo et al., 2018a), whose gametophytes are also achlorophyllous (Bierhorst, 1971b). Here we found that in Schizaeaceae, there were actually two independent losses of *chl* genes (Figures 1, 2; Supplementary Figure 2). One of them, however, is found in *S. pusilla*, a species that produces chlorophyllous gametophytes (Figures 1, 2). The link between *chl* gene loss and the achlorophyllous nature of gametophytes might therefore seem not obligate. The gametophytes of *S. pusilla* likely rely on nuclear-encoded light-dependent NADPH-protochlorophyllide oxidoreductase (LPOR) instead, the alternative pathway for chlorophyll synthesis, but advanced genetic evidence is required to test such a hypothesis. On the other hand, several fern and lycophyte lineages producing achlorophyllous gametophytes retain functional *chl* genes in their plastomes, such as Lycopodiaceae, Ophioglossaceae, and the *Schizaea* clade in the present case (reviewed in Kuo et al., 2018a). These retentions imply that DPOR remains important for chlorophyll synthesis in the autotrophic (or mixotrophic) sporophytes of these ferns. Alternatively, DPOR could be involved in different physiological functions, resembling the situation in the (potentially) fully mycoheterotrophic bryophytes, which also have *chl*-retained plastomes (Bell et al., 2020).

In addition to *ndh* and *chl* genes, several other coding and tRNA genes have disappeared from Schizaeaceae plastomes (Figures 1, 2). From our blast results against the transcriptomes, the coding genes are unlikely to have been transferred to the nuclear genome. However, these genes do not seem to be associated with the specialization of gametophytic lifestyle in Schizaeaceae, and are mostly considered to have minor functions in the plastid. For instance, *rps16* has been lost several times in ferns (Zhang et al., 2014; Kuo et al., 2018a; Du et al., 2022). *Ycf94*, which was recently identified in plastomes of seed-free plants and whose function is still unknown (Song et al., 2018), seems to be absent only in Schizaeaceae (Du et al., 2022).

### Conclusion and Future Perspectives

Schizaeaceae is one of the most understudied families of ferns. With the most comprehensive sampling to date, our phylogenomic analyses resolved important relationships in this family, and provided the most robust infrafamiliar backbone for Schizaeaceae. We proposed a new phylogenetic classification modified from the Reed’s (1947) system, in which a portion of *Schizaea sensu* PPG I (2016) is moved to a third genus—*Microschizaea*. In addition, our species-level phylogeny illustrated species complexes within this family. One cryptic species hidden in the *S. dichotoma* complex was identified and described as a new species, *Schizaea medusa*. Using our new phylogenetic framework, we were able to trace the evolution of plastome features as well as the gametophytic lifestyle in Schizaeaceae. Specifically, we provided a better picture of the relationship between plastid gene loss and mycoheterotrophy in fern gametophytes.

Importantly, this phylogenetic study sets the stage for future work delving into the unique biological features of Schizaeaceae and in Schizaeales, such as Mesozoic biogeography (Skog, 2001), epiphytism on tree ferns (Amoroso et al., 2020), leaf simplification and lamina reformation (Vasco et al., 2013), biosynthesis of silica bodies (Ribeiro et al., 2007; Tzu-Tong Kao personal communications), and recruitment of symbiotic microbiome (e.g., Chen et al., 2022). Lastly, the pheromone-mediated (i.e., antheridiogen) mating system has been well-studied in the other two Schizaeales families (Yamane, 1991, 1998; Tanaka et al., 2014) but not yet for Schizaeaceae. It would be very interesting to explore whether gametophytes of Schizaeaceae rely on the similar pheromone system for their “underground” mating.

## Collaborators of GoFlag Consortium

GoFlag is an NSF-funded project (DEB 1541506) based at the University of Florida, Field Museum, and University of Arizona. Project personnel include (at UF): J. Gordon Burleigh, Emily Sessa, Stuart McDaniel, Christine Davis, Pavlo Antonenko, Sarah Carey, Lorena Endara, Weston Testo; (at Field): Matt von Konrat, Eve Gaus; (at UA): Hong Cui.

## Data Availability Statement

The datasets presented in the study are publicly available. The data can be found at Nucleotide Collections at GenBank: https://www.ncbi.nlm.nih.gov/nuccore/ with the accession numbers: ON120846, ON207049-ON207054, ON314247-ON314248, and ON368093-ON368187.

## Author Contributions

L-YK and B-FK designed the experiments and drafted the manuscript. L-YK, B-FK, GoFlag Consortium, and F-WL carried out the experiments. L-YK, B-FK, and G-JW analyzed the data. PL, GR, C-WC, LS, DO, MR, and KK collected important samples and DNA sequences. L-YK, B-FK, GR, and F-WL collected materials for the taxonomic treatment. All authors contributed to the article and approved the submitted version.

## Funding

The main funding was from Ministry of Science and Technology of Taiwan (MOST 109-2621-B-007-001-MY3), NSF GoLife program (DEB-1541506 to P Antonenko, JG Burleigh, EC Davis, SF McDaniel, and EB Sessa), and the Bioresource Conservation Research Center in College of Life Science from the Higher Education Sprout Project by MOE was granted.

## Conflict of Interest

G-JW and MR were employed by Taiwan Semiconductor Manufacturing Company and Wildland Consultants.

The remaining authors declare that the research was conducted in the absence of any commercial or financial relationships that could be construed as a potential conflict of interest.

## Publisher’s Note

All claims expressed in this article are solely those of the authors and do not necessarily represent those of their affiliated organizations, or those of the publisher, the editors and the reviewers. Any product that may be evaluated in this article, or claim that may be made by its manufacturer, is not guaranteed or endorsed by the publisher.
