# Supplementary figures and images for: Systematics and Plastome Evolution in Schizaeaceae
Source: Front Plant Sci. 2022 Jul 13;13:885501. doi: 10.3389/fpls.2022.885501 (PMC9328107; doi:10.3389/fpls.2022.885501)

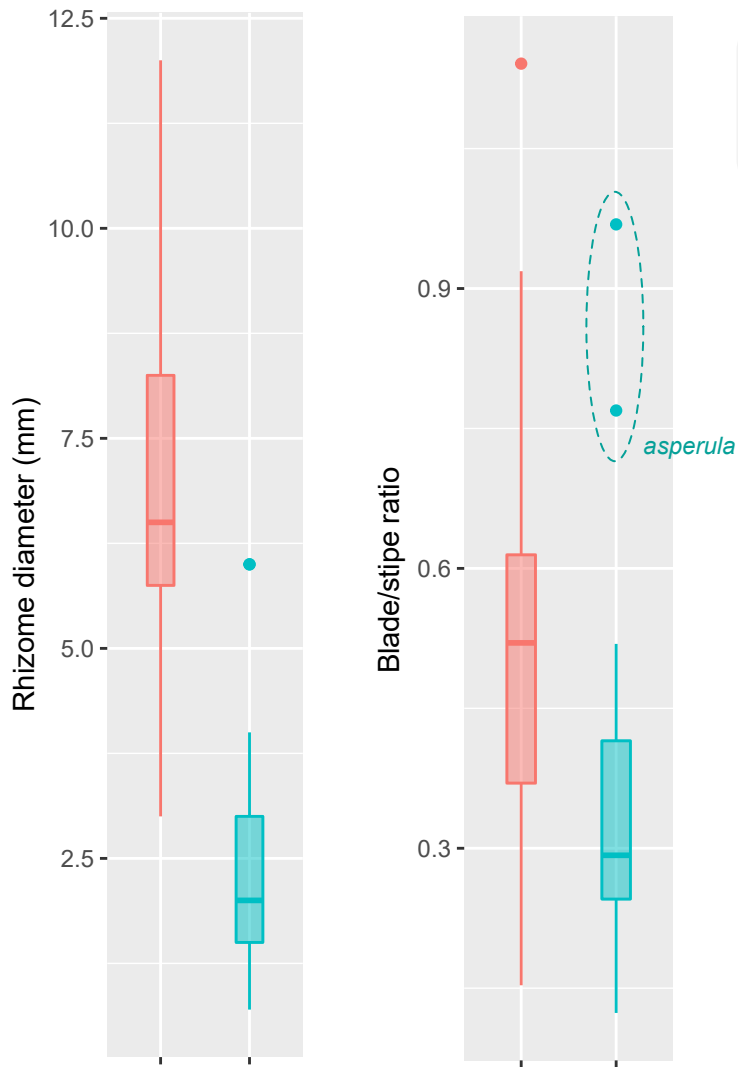

Supplement: Supplementary file 5 [file Image_1.pdf]
